# Effects of trauma mattress on dose and image quality of paediatric whole-body computed tomography examinations

**DOI:** 10.1186/s12880-025-01821-y

**Published:** 2025-07-06

**Authors:** Jacob Leonard Ago, Stephen Inkoom, Benard Ohene-Botwe, Alise Larsen, Ingerd Skaaret Berg

**Affiliations:** 1 Department of Radiography, School of Biomedical and Allied Health Sciences, College of Health Sciences, University of Ghana, Korle-Bu Campus, Korle-Bu, P. O. Box KB 143, Accra, Ghana; 2 Department of Nuclear Safety and Security, School of Nuclear and Allied Sciences, College of Basic and Applied Sciences, University of Ghana, Atomic Campus, Accra, Ghana; 3 Department of Medical Physics, School of Nuclear and Allied Sciences, College of Basic and Applied Sciences, University of Ghana, Atomic Campus, Accra, Ghana; 4 Radiation Protection Institute (RPI), Ghana Atomic Energy Commission, Accra, Ghana; 5 Department of Midwifery and Radiography, School of Health & Medical Sciences, City, University of London, London, UK; 6 Medical Physics Unit, Innlandet Hospital, Hamar, Norway

**Keywords:** Whole-body computed tomography, Trauma mattress, Immobilising devices, Bearing devices, Anthropomorphic Phantom, Dose optimisation

## Abstract

**Background:**

Whole-body computed tomography (WBCT) is the preferred first line investigation for patients with suspected multiple traumas. To decrease the potential for increased spinal injury, bearing devices, including trauma mattress, are recommended for adequate spine immobilisation. This study assesses the effect of trauma mattress on the dose and image quality of WBCT examinations.

**Methods:**

This was a phantom-based experimental study. Two different paediatric whole-body anthropomorphic phantoms from Kyoto Kagaku were used: newborn (PBU-80) and 5-year-old (PBU-70). Optimised WBCT protocols were scanned with and without a trauma mattress. The effective dose (ED) from each protocol was estimated from CT-Expo software and from the product of the dose length product and dose conversion coefficient (DLP-E(k)) methods, while image quality was assessed subjectively and objectively.

**Results:**

The use of trauma mattress increased the mean ED and decreased the SNR of the 5-year-old phantom examinations by 7.0% (*p* = 0.776) and 21.4% (*p* = 0.194) respectively. In contrast, there was a 43.9% increase in ED (*p* = 0.019) and a 16.5% decrease in SNR (*p* = 0.221) when trauma mattress was used for the newborn phantom examinations. The differences in the mean ED from CT-Expo and the DLP-E (k) were not statistically significant (*p* = 0.258 and 0.278 for newborn and 5-year-old phantoms, respectively). The median organ doses estimated from all examinations performed without a trauma mattress were significantly lower than examinations performed with a trauma mattress (*p* = 0.001). The use of the trauma mattress increased the average tube voltage, tube current, volume computed tomography dose index (CTDI_vol_), and the dose-length product (DLP) by 1.3%, 63.9%, 48.3%, and 47.3%, respectively. However, a significant increase was only observed in the tube current (*p* = 0.014).

**Conclusion:**

The use of trauma mattress increased the ED and decreased the SNR during the WBCT examinations, albeit at different levels for the newborn and 5-year-old phantoms. Consequently, medical imaging professionals should restrict the use of bearing devices to examinations that justifiably require them. Appropriate adjustments in scan protocols for different body habitus and the use of alternative immobilisation techniques, where necessary, will further enhance patient safety during paediatric WBCT examinations.

## Introduction

Whole-body computed tomography (WBCT) is a commonly used computed tomography (CT) technique for assessing patients with suspected multiple traumas, involving injuries to multiple body parts [1–5]. WBCT combines noncontrast-enhanced examination of the head and neck with a contrast examination of the trunk, including the whole spine [2, 3]. It is highly accurate and can effectively detect life-threatening injuries with good sensitivity and specificity, thereby improving patient management [6]. Although this, along with its rapid results generation, has increased demand for treating trauma patients where time is crucial, the increased use of WBCT has raised concerns about radiation-induced health problems due to the high levels of exposure [7, 8].

The possibility of detrimental effects associated with WBCT may be greater in paediatric population due to their increased radiosensitivity and longer life expectancy [9]. During WBCT imaging, bearing devices such as trauma mattress are used to support and ensure adequate immobilization of the spine to decrease the potential for increased spinal injury [10]. However, reports suggest that the use of these devices may affect the radiation dose and image quality, particularly signal relative to noise [10, 11]. To the best knowledge of the authors, the effects of the use of trauma mattress on radiation dose and image quality during paediatric WBCT examinations are less understood.

This study assessed the effect of using trauma mattress on the dose and image quality of WBCT examinations from two different CT scanners. The purpose is to understand the extent to which bearing devices, such as trauma mattresses, impact WBCT regarding dose and image quality and to make appropriate recommendations to support and optimize their use in newborns and paediatrics.

## Materials and methods

### Materials

Two CT scanners, anthropomorphic phantoms, trauma mattress, and CT-Expo software were the materials used for the study. In addition, a Black Piranha 657 (B2-16020228, RTI, Sweden) and Catphan 500 were used to undertake quality control activities on both scanners, following the international elctrotechnical commission (IEC) guidelines [12].

**Fig. 1:**
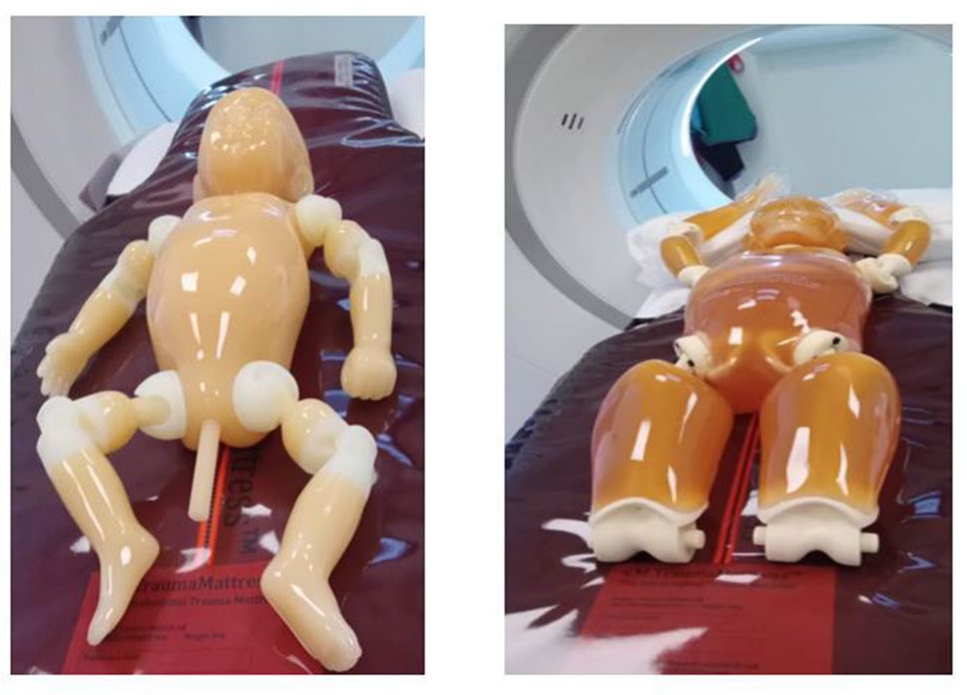
Newborn (L) and 5-year-old (R) anthropomorphic phantoms positioned on CM trauma mattress

The Kyoto Kagaku newborn (PBU-80) and 5-year-old (PBU-70) whole body anthropomorphic phantoms (Kyotokagaku America Inc., Los Angeles, United States of America) (Fig. 1) [henceforth newborn phantom and 5-year-old phantoms, respectively] have soft tissues made with urethane-based resin and synthetic bones made with epoxy resin. Both the newborn and 5-year-old phantoms have no metal parts or liquid structures. The newborn phantom has a size of 53 cm, a weight of 3.5 kg, a packaging size of W57 X D44 X H29 cm, and a packaging weight of 8 kg. Conversely, the 5-year-old phantom has an HU number approximate to the human body and its main joints are close to human articulation. It has a height of 110 cm, a weight of 20 kg, and a packaging size of W86 X D60 X H32 cm. The newborn and 5-year-old phantoms are, in terms of dimensions, comparable to the infant and child mathematical phantoms, respectively, in CT-Expo.

The CM TraumaMattress™ (Comfort Medical AB, Sweden) (Fig. 1) is a CT-compatible intrahospital mattress that helps in the immobilisation and transfer of patients. The mattress is made of polyester-weave that can be removed and a silicone-coated polyamide with low friction attributes that lies under the mattress. The core of the mattress is waterproof and is covered in a durable polyurethane-cover [13]. The CM trauma mattress weighs 8 kg in weight and measures 2000 × 530 × 60 mm in dimension.

The General Electric (GE) Revolution^™^ CT (GE Medical Systems, Waukesha, United States of America) and the Siemens SOMATOM Definition Edge CT scanners (Siemens Healthineers, Erlangen, Germany) were used for this study. The GE Revolution® CT is a 256 MDCT equipment with in-built Smart technologies, which help to produce high quality images at lower radiation dose levels in the examination of various disease conditions including polytrauma and/or WBCT. The Siemens SOMATOM Definition Edge is a 128 MDCT equipment with an in-built Advanced Modelled Iterative Reconstruction (ADMIRE) technology. Both the GE and Siemens scanners have automatic attenuation-based kilo-voltage peak (kVp) selection algorithms that produce the lowest radiation dose with 70, 80, 100, 120, or 140 kVp [14, 15]. The reconstruction algorithms used for the GE and Siemens scanners were the Deep Learning Image Reconstruction (DLIR) with the “high” strength level and the Advanced Modeled Iterative Reconstruction (ADMIRE) strength 3, respectively.

The CT-Expo™ software version 2.5 (SASCRAD, Fritz-Reuter-Weg, Buchholz, Germany) [16] was used to estimate the effective dose (ED) for all protocols and the organ doses associated with the optimized protocols. The infants (BABY) and 7-year-old children (CHILD) models were used in this study.

### Quality control checks

We conducted status checks in accordance with the International Electrotechnical Commission’s (IEC) International Standard on “Evaluation and routine testing in medical imaging departments–part 3–5: acceptance and constancy tests–imaging performance of computed tomography X-ray equipment”. Both scanners were air-calibrated the same day before measurements were taken. A helical imaging was performed over the entire Catphan 500 with a standard helical protocol. The QC tests undertaken were dose measurement free in air, CT number, homogeneity, and noise. Results for all tests performed were within tolerance limits and are available upon reasonable request.

### CT examinations

The whole-body examinations were performed in two parts: non-contrast examination of the head (in head-first supine position) and contrast-enhanced examination of the chest-abdomen-pelvis (CAP) (in feet-first supine position). The protocols used for these examinations were developed through dose and image quality optimisation using the figure-of-merit approach, and the results have been published elsewhere [17]. In this study, the optimised protocols for the arterial phase CAP were used (Table 1) as all examinations for the head were performed without trauma mattress. The pitch and slice thickness for each of the optimised protocols were kept constant throughout the data collection for both scanners and phantom sizes. Solid inserts were used to simulate the effect of contrast media, as this was a phantom study. Twenty-seven different examinations were performed (9 without and 18 with the trauma mattress) and tested for dose and image quality. The table height was adjusted to be in isocentre for each examination, both with and without trauma mattress, to prevent possible displacement.

**Table 1:** Scan protocols. For full set of optimised protocols, see ago et al. [17]

Phantom	Scan Mode	Noise Index/	Auto- Prescription/ Quality Ref. mAs	Pitch	Tube Voltage [kVp]	Tube Current [mA]	Rotation time [s]	Total Collimation [mm]	Table feed [mm]	Slice thickness [mm]
GE Scanner										
Newborn	Axial	18	Off	1.00	80	150	0.28	80.00	80.00	0.625
5-year-old	Helical	26	On	0.52	100	98.79	0.28	40.00	20.80	0.625
Siemens Scanner										
Newborn	Helical	-	740	1.400	80	129.0	0.50	38.40	53.76	0.75
5-year-old	Helical	-	662	1.400	80	306.00	0.50	38.40	53.76	0.75

Key: Autoprescription = GE scanner; Quality Reference mAs = Siemens scanner

### Effective dose estimation

In estimating the ED from CT-Expo software, the BABY and CHILD modules were selected for the newborn and 5-year-old phantoms, respectively. In addition, both male and female genders were selected in turns while estimating the effective and organ doses. Tissue weighting factors as stated in ICRP publication 103 were used in estimating the effective doses. The scan lengths used in estimating the ED are presented in Table 2. Additionally, the ED was estimated from a simplified method according to Eq. (1) [18].

**Table 2:** Scan lengths and k-values used in dose calculation from CT-Expo and DLP-E(k) methods respectively

	Scan Range				Scan Length		k-values^15^
	From z-		To z+		L (cm)		
	Male	Female	Male	Female	Male	Female	
Newborn Chest	14	11	25	22	11	11	0.099
Paediatric Chest	26	24	43	40	17	16	0.047
Newborn Abdomen-Pelvis	0	1	18	18	18	18	0.092
Paediatric Abdomen-Pelvis	0	1	28	27	28	26	0.043
Newborn Trunk	1	1	23	22	22	21	0.086
Paediatric Trunk	0	1	41	40	41	39	0.041

1 $$\:ED=kDLP$$

Where *k* is the conversion coefficient specific to each body part. The *k*-factors used in this study were adopted from Romanyukha et al. [19].

### Subjective image quality assessment

The images were subjectively assessed by two independent radiologists, with 5 years’ and 8 years’ experience, for artefacts, image noise, contrast, organ visibility, and overall diagnosability. This was done in a blinded fashion by removing all annotations related to scan protocols and organised randomly in order to decrease expectation bias. Each category was assessed using a 5-point Likert scale (Table 3) adapted from Gariani et al. [20]. Inter-rater agreement between the two radiologists was assessed with the Cohen’s kappa coefficient [21]. Additionally, the assessments were combined, and the average scores computed to determine the effect of the trauma mattress on the five image quality criteria.

**Table 3:** Five-point likert scale for subjective image quality assessment

Parameter	Scale				
	1	2	3	4	5
Artefacts	Severe unacceptable artifacts	Moderate artifacts, partially degrading diagnostic capability;	Slight artifacts, not significantly affecting diagnostic capabilities;	Minimal artifacts	Absent, no perceivable artifacts
Image Noise	Bad, not diagnostic	Poor, definitely noisy	Moderate, slightly noisy but acceptable	Minimal noise, not affecting diagnostic quality	No perceivable noise.
Contrast	Non-diagnostic image quality	Severe blurring with uncertainty about the evaluation	Moderate blurring with restricted assessment	Slight blurring with unrestricted diagnostic image evaluation possible	Excellent image quality
Organ visibility	Very poor, almost not visible	Poor	Intermediate	Good	Excellent
Overall diagnosability	Bad, not diagnostic	Poor, diagnostic confidence substantially reduced	Moderate, sufficient for diagnosis	Good	Excellent

### Objective image quality assessment

**Fig. 2:**
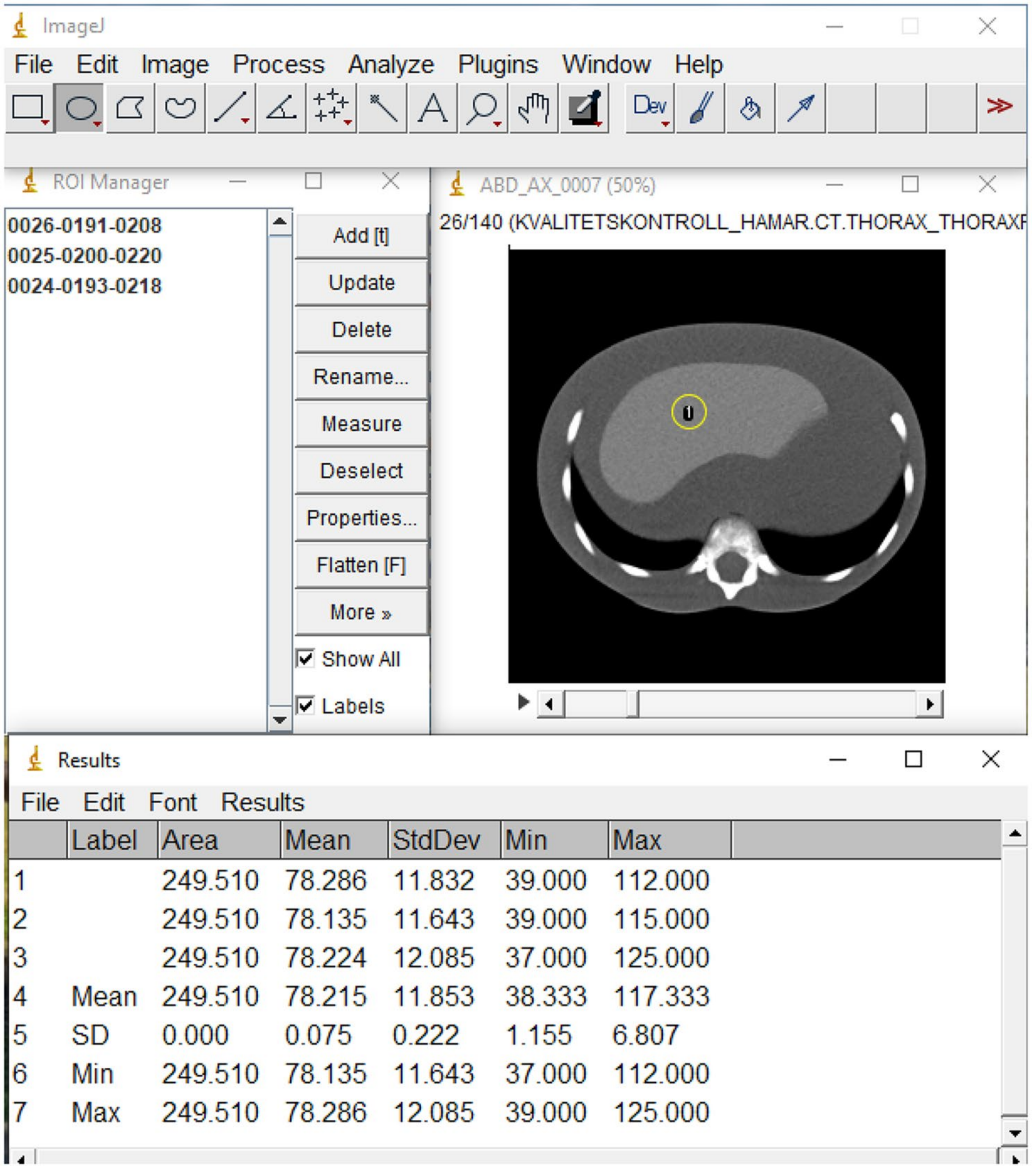
ROI for SNR determination from ImageJ software

Quantitatively, image quality was assessed using the ImageJ software version 1.54c (Wayne Rasband and Contributors, National Institutes of Health, USA). The signal-to-noise ratio (SNR) was used in the objective assessment of the image quality. In determining the SNR for body examinations, three circular regions of interest (ROIs) of equal dimensions (5.0 mm x 5.0 mm) were drawn on three different images of the liver (for the 5-year-old phantom) and heart (for the newborn phantom) at the same slice position. Figure 2 shows how the signal and standard deviations (noise) were obtained from the ImageJ. The average values of the signal and standard deviations obtained were used to calculate the SNR according to Eq. (2) [20].

2 $$\:SNR=\frac{Mean\:Signal\:in\:Roi}{Standard\:Deviation\:in\:ROI}$$

### Data analysis

The data obtained was statistically analysed with the IBM SPSS v26.0 (IBM Corp., Armonk, N.Y., USA) and Microsoft Excel 2016. Results from descriptive statistics were reported as the mean ± standard deviation for normally distributed data and median (95% confidence interval) for non-normally distributed data and presented in the form of tables and graphs. The Shapiro-Wilk test was used to determine the normality of the data. Normally distributed data sets were compared using the one-way ANOVA test whereas comparison between non-normally distributed data sets was made with the Mann-Whitney U-test and Kruskal-Wallis H test. Differences were considered significant at *p* < 0.05.

## Results

### CT scan parameters

The average tube voltage increased marginally by 1.3% (*U* = 96.50, *p* = 0.897) whereas the average tube current increased significantly by 63.9% (*U* = 42.00, *p* = 0.014) when trauma mattress was used. Additionally, the use of the trauma mattress increased both the CTDI_vol_ and DLP by 48.3% and 47.3% respectively, although these were not statistically significant (*U*,* p* = 65.00, 0.139; 70.00, 0.207, respectively). Similarly, there were non-significant increase in the pitch (*U* = 71.50, *p* = 0.213), slice thickness (*U* = 85.00, *p* = 0.452), and rotation time (*U* = 78.00, *p* = 0.315) when a trauma mattress was used. These results have been presented in Table 4.

**Table 4:** Average scanning parameters used for examinations performed with and without the trauma mattress

Parameter	Mean ± Standard deviation		Significance
	Without trauma mattress	With trauma mattress	U (*p*-value)
Tube voltage [kVp]	84.44 ± 8.31	85.56 ± 10.66	96.50 (0.897)
Tube current [mA]	129.65 ± 65.16	212.51 ± 102.34	42.50 (0.014)
Rotation time [s]	0.34 ± 0.09	0.37 ± 0.10	78.00 (0.315)
Slice thickness [mm]	0.65 ± 0.05	0.67 ± 0.06	85.00 (0.452)
Pitch	0.82 ± 0.36	1.00 ± 0.31	71.50 (0.213)
CTDI_vol_ [mGy]	1.51 ± 0.88	2.24 ± 1.19	65.00 (0.139)
DLP [mGy.cm]	48.45 ± 38.03	71.36 ± 50.17	70.00 (0.207)

### Subjective image quality assessment

Cohen’s Kappa was used to determine the interobserver reliability of the subjective image quality assessment by the two observers for examinations performed with and without the trauma mattress. The Cohen’s Kappa score obtained for examinations performed without trauma mattress ranged from 0.65 to 0.81 for newborn phantom examinations and 0.62 to 0.89 for the 5-year-old phantom examinations. In contrast, examinations performed with the trauma mattress had Kappa score from 0.60 to 0.79 for newborn phantom examinations and 0.61 to 0.70 for the 5-year-old phantom examinations (Table 5). These results indicate a weak to excellent agreement between the two observers [21].

**Table 5:** Subjective image quality assessment scores and interobserver reliability for examinations performed with the trauma mattress

	With Trauma Mattress				Without Trauma Mattress			
**Image Quality Parameters**	**Average Ratings**		**Kappa Score**	***p*** **-Value**	**Average Ratings**		**Kappa Score**	***p*** **-Value**
	**Reader 1**	**Reader 2**			**Reader 1**	**Reader 2**		
**Newborn phantom examinations**								
Artifact	2.72	2.71	0.79	< 0.001	3.14	3.07	0.81	< 0.001
Image Noise	3.31	3.20	0.68	< 0.001	3.44	3.41	0.79	< 0.001
Image Contrast	3.24	3.07	0.60	< 0.001	3.34	3.12	0.77	< 0.001
Organ Visibility	3.22	3.18	0.75	< 0.001	3.42	3.31	0.76	< 0.001
Overall Diagnosability	3.29	3.27	0.71	< 0.001	3.37	3.24	0.65	< 0.001
**5-year-old phantom examinations**								
Artifact	2.91	2.73	0.63	0.002	3.00	2.94	0.89	< 0.001
Image Noise	3.55	3.55	0.69	< 0.001	3.38	3.13	0.62	< 0.001
Image Contrast	3.44	3.25	0.70	< 0.001	3.45	3.27	0.62	0.026
Organ Visibility	3.06	2.94	0.70	< 0.001	3.45	3.55	0.83	0.001
Overall Diagnosability	3.25	3.13	0.61	< 0.001	3.64	3.45	0.69	0.001

**Fig. 3:**
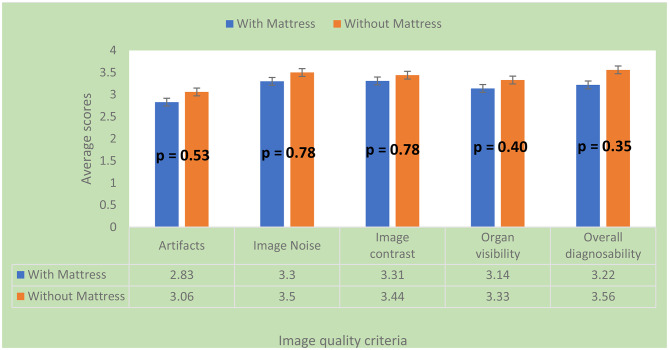
Bar chart showing the differences in subjective image qualities between examinations performed with and without trauma mattress

**Fig. 4:**
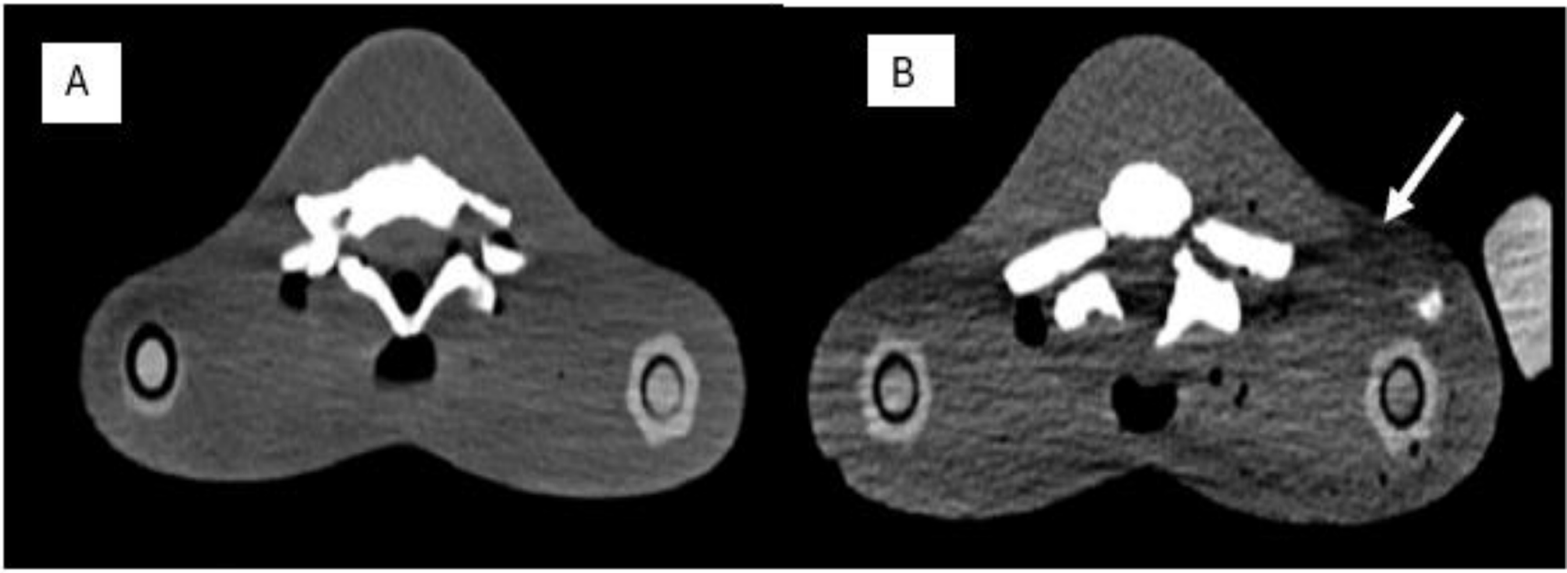
**A**: Examination without trauma mattress; **B**: Examination with trauma mattress; White arrow: Streaking/beam hardening artefact

The examinations performed without the trauma mattress (*n* = 9) had higher mean ratings than examinations performed with the trauma mattress (*n* = 18) for all the image quality criteria for subjective assessment (Fig. 3). However, a Mann-Whitney U test revealed no statistically significant difference across all criteria (Fig. 3). The main type of artefact found by the two radiologists was streaking artefacts (Fig. 4).

### Comparison of SNR between examinations performed with and without trauma mattress

The mean SNR of examinations performed without a trauma mattress was 16.5 and 21.4% higher than examinations performed with a trauma mattress for the newborn and 5-year-old phantoms, respectively (Table 6). A Mann-Whitney U-test showed that the difference in the mean SNR of examinations performed with and without trauma mattress was not statistically significant for both the newborn phantom (*U* = 16.00, *p* = 0.221) and 5-year-old phantom (*U* = 5.00, *p* = 0.194) examinations.

**Table 6:** SNR and ED of newborn and paediatric trunk examinations with and without trauma mattress

Examination	(*n*) Mean ± Standard Deviation			
	Signal-noise-ratio		Effective dose (mSv)	
	With Trauma Mattress	Without Trauma Mattress	With Trauma Mattress	Without Trauma Mattress
Newborn Trunk	(*n* = 11) 1.67 ± 0.65	(*n* = 5) 2.00 ± 0.48	(*n* = 11) 2.53 ± 1.31	(*n* = 5) 1.42 ± 0.53
Paediatric Trunk	(*n* = 8) 5.52 ± 1.85	(*n* = 3) 7.02 ± 1.42	(*n* = 8) 3.84 ± 0.70	(*n* = 3) 3.57 ± 0.81

### Comparison of ED between examinations performed with and without trauma mattress

The mean EDs for the examinations performed with trauma mattress were 43.9% and 7.0% higher than the corresponding values obtained from examinations performed without trauma mattress for the newborn and 5-year-old phantoms respectively (Table 6). A Mann-Whitney U test showed a statistically significant difference for examinations performed with the newborn phantom (*U* = 7.50, *p* = 0.019) whereas the difference was not statistically significant for examinations performed with the 5-year-old phantom (*U* = 10.00; *p* = 0.776).

### Comparison of ED estimated from CT-Expo and DLP-E(k) methods

**Fig. 5:**
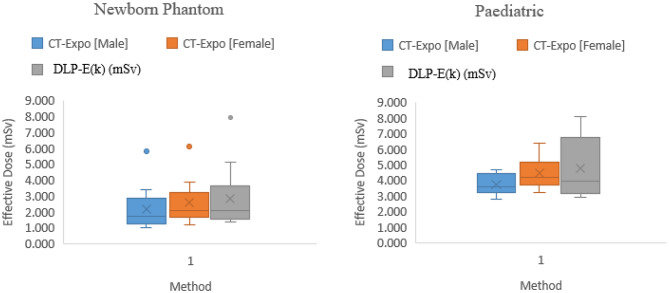
Box plots of effective doses from newborn phantom (left) and paediatric phantom (right) estimated from CT-Expo and DLP-E (k): *the X in the box represents the mean effective doses while the midline in the box indicates the median effective doses*,* the T-bars that extend from the box denote the minimum and maximum effective doses from each method (without outliers)*,* and the box represents the 25th to 75th percentile.* Outliers as seen in the newborn phantom images could be due to non-human equivalent tissues used for this phantom

The median ED estimated from CT-Expo for all newborn phantom examinations was 1.65 mSv (95% CI = 1.49 to 2.67 mSv) and 2.05 mSv (95% CI = 1.81 to 3.05 mSv) for male and female anatomies, respectively, whereas the median effective dose for all newborn phantom examinations estimated from DLP-E (k) is 2.10 (95% CI = 1.90 to 3.59 mSv). A Kruskal-Wallis H test showed no significant difference in effective dose between the methods, $\:{\upchi\:}$^2^(2) = 2.706, *p* = 0.258, with a mean rank effective dose of 22.61 mSv, 29.14 mSv, and 30.75 mSv for CT-Expo (Male), CT-Expo (Female), and DLP-E (k) respectively. For the 5-year-old phantom examinations, a one-way ANOVA revealed that the mean effective dose from DLP-E (k) [4.39 ± 2.02 mSv] was not significantly higher than the effective doses from CT-Expo (Male) [3.35 ± 1.20 mSv] and CT-Expo (Female) [3.96 ± 1.59 mSv], (F(2, 36) = 1.325, *p* = 0.278). Figure 5 shows the relationship between the effective doses estimated from CT-Expo with male anatomy [CT-Expo (Male)], CT-Expo using female anatomy [CT-Expo (Female)], and DLP-E (k) methods for the newborn and paediatric phantom examinations.

### Estimated organ doses

The testis received estimated doses of 1.05 and 2.10 mSv from newborn phantom protocols without and with trauma mattress, respectively. As well, estimated doses for newborn phantom protocols with and without trauma mattress were 4.3 mSv and 2.45 mSv, respectively for each of bladder, kidneys, ovaries, prostate, and uterus. In contrast, the oesophagus received estimated organ doses of 4.05 mSv and 4.30 mSv for the 5-year-old phantom examinations performed without and with trauma mattress, respectively. It was also found that the breast received a dose of 4.95 mSv and 4.05 mSv for the 5-year-old phantom examinations performed with and without trauma mattress. As well, the kidneys received estimated doses of 6.7 mSv and 5.3 mSv for the 5-year-old phantom examinations performed with and without trauma mattress. The estimated doses for the testis for the 5-year-old phantom examinations without and with trauma mattress were 4.2 mSv and 4.65 mSv, respectively, while those for the ovaries were 3.7 mSv and 4.4 mSv, respectively. All newborn phantom examinations performed without a trauma mattress had a median organ dose of 2.40 mSv (95% C.I = 2.01 to 2.42 mSv). This was significantly lower than the organ doses from all newborn phantom examinations performed with the trauma mattress, median = 4.15 mSv (95% C.I = 3.22 to 4.06 mSv) [*U* = 15.50, *p* < 0.001]. Similarly, the median organ dose for all 5-year-old phantom examinations performed without the trauma mattress (median = 4.48 mSv; 95% C.I = 4.36 to 4.49 mSv) was significantly lower than the organ doses for all the 5-year-old phantom examinations performed with the trauma mattress, median = 7.15 mSv (95% C.I = 5.77 to 7.14 mSv) [*U* = 29.50, *p* < 0.001].

## Discussion

This phantom-based experimental study throws light on the effects of the use of trauma mattress during paediatric WBCT examinations, and whether there is a need for the use of trauma mattress during paediatric WBCT examinations. It is known that the use of WBCT examinations is associated with high radiation doses as compared to conventional X-ray examinations. However, WBCT examinations are essential in the event of poly-trauma as they potentially increase the survival rates of poly-traumatised patients [22]. Despite the level of dose associated with WBCT, studies relating to how the use of bearing devices affects the dose and image quality are scarce [11]. Neglecting the effects of these devices on the dose and image quality during WBCT examinations, especially for the paediatric population, may be detrimental.

This study found a mean ED of 1.42 ± 0.53 mSv for CAP examinations performed without a trauma mattress with the newborn phantom. This ED increased by 43.87% when the examination was performed with a trauma mattress, along with a statistically significant difference (*p* = 0.019). In contrast, this study found a mean ED of 3.57 ± 0.81 mSv for CAP examinations performed without a trauma mattress for the 5-year-old phantom, with a corresponding 7.03% non-significant increase when the examination was performed with a trauma mattress (*p* = 0.776). These differences could be due to the different sizes and compositions of the phantoms, reflecting that different body habitus may be affected to varying degrees when a trauma mattress is used in WBCT examinations. Table 6 shows the difference in ED between examinations performed with and without a trauma mattress. The result from this study aligns with the findings from a study by Loewenhardt et al. [23] where the use of different immobilising devices caused an increase in ED from 2.5 to 4.5%. Similarly, Euler et al. [24] reported that the use of immobilising devices causes an increase in radiation dose. Stokkeland et al. [10] also remarked that denser devices result in a lesser increase in ED. The increased ED estimated from DLP-E(k) method confirms the results from previous studies [18, 25] that the simplified method overestimate the ED at different levels for different body parts and sizes. A more comprehensive dosimetric approach through direct dose measurements using thermoluminescent dosimeters placed inside the phantom to quantify absorbed dose may help correct any inherent effect of the trauma mattress (through attenuation by the trauma mattress materials) on the estimated dose.

The increase in ED may vary for different protocols and equipment designs. Additionally, different bearing devices may have differing effects on the extent of the increase in ED. For the different devices used in their study, Stokkeland et al. [10] reported that the highest increase in ED occurred with vacuum mattress and concluded that it may have resulted from increased photon attenuation. In a similar study, rigid spine board with soft foam headblocks increased the ED of a trauma CT examination by 1.7–3.4 mSv [11]. Furthermore, the increased estimated EDs for female against males aligns with the result of a similar study [26] and could be due to anatomical differences between males and females. Again, our findings align with the study by Dimitroukas et al. [26] which reported a slight increase in ED estimation using k-factors against Monte Carlo-based software (VirtualDoseCT) similar to CT-Expo.

The organ doses for the trunk estimated from this study were lower than those estimated in a study by Gao et al. [27]. The authors [27] found the dose to the male gonads for age group < 1 year to be 3.2 mSv against 2.10 mSv from this study. For paediatric examinations, Gao et al. [27] estimated the organ dose to the male and female gonads to be 9.9 and 8.5 mSv respectively against 4.2–4.65 mSv and 3.7–4.4 mSv to testis and ovaries respectively from this study.

In addition to increasing the radiation dose, the use of bearing devices may decrease the image quality when used in WBCT examinations. The rate at which bearing devices may decrease the image quality may differ based on the protocol, the size of the patient, the equipment design, and the type of bearing device. In this study, the objective image quality (SNR) of examinations conducted without the use of a trauma mattress showed improvements of 16.5% and 21.3% for the newborn and 5-year-old phantoms, respectively, compared to examinations performed with a trauma mattress. This could be due to the difference in tissue composition for the newborn (with non-human equivalent tissues) and 5-year-old phantoms (with human equivalent tissues).

This result is similar to that of Euler et al. [24], which reported that the use of immobilising devices caused a decrease in image quality. In order to obtain images of comparable quality, Hemmes et al. [11] increased the exposure by 11% when studying the effects of bearing devices on the dose and image quality of trauma CT examinations with rigid spine board and soft foam headblocks. The bearing device used for this study is CT compatible and has low X-ray attenuating properties [13]. This could have resulted in the non-significant differences in the results (ED and SNR) of examinations performed with and without the trauma mattress. As well, the non-significant differences could be due to the strengths of the reconstruction algorithms used [28].

This study also found that the use of trauma mattress resulted in increased image artefacts and noise, and decreased contrast, organ visibility, and overall diagnosability. Consistent with a similar study [10], the main artefact found in this study for examinations performed with trauma mattress was beam hardening/streaking artefacts, although these did not significantly reduce the clinical relevance of the images. Loewenhardt et al. [23] also observed that some immobilising devices caused massive artefacts. Quantitatively, Euler et al. [24] reported a 6.6–11.2% increase in image noise following the use of immobilising devices.

### Limitation

The study did not include results for the head CT examinations because they were all performed with trauma mattress and hence did not permit comparison to be made. All reported results are related to CAP WBCT protocols. As well, the non-human equivalent tissues used for the newborn phantom (resulting in outliers seen in Fig. 5 as any small differences in anatomy could lead to relatively large differences in dose estimates) limited the extent of objective IQ analysis as only SNR could be performed. This study did not explore the performance of each of the two CT scanners and their effect on dose and image quality. It is advised that future studies should consider this to provide insight for potential scanner-specific protocol optimisation during WBCT with trauma mattress. Moreover, the small sample sizes used for this study means limit the generalisability of the results. Thus, further study is required to verify the findings with larger sample sizes.

## Conclusion

The use of trauma mattress increased the ED and reduced objective image quality in the 5-year-old phantom examinations as well as the objective image quality for the newborn phantom examinations, albeit these changes were not statistically significant. In contrast, the use of trauma mattress resulted in a significant increase in EDs for the newborn phantom examinations. Additionally, the use of trauma mattress resulted in a significant increase in organ doses. Nonetheless, the relevance of bearing devices in paediatric trauma CT cannot be underestimated, as they reduce the possibility of increasing secondary spinal injuries and decrease hospital stay times. While designers of bearing devices may consider the radiological properties of the materials used, it is vitally important for radiological professionals to consider the patient’s clinical history and body habitus and vary the exposure factors accordingly to ensure appropriate justification for the use of bearing devices, where necessary, is maintained, and to enhance the radiation protection of the radiosensitive organs. This is important given the increase in dose parameters and observed reduction in image quality, especially for the newborn examinations. Therefore, restricting the use of bearing devices to examinations that justifiably require them will ensure the full benefits of bearing devices are obtained. Where necessary, medical imaging professionals should employ alternative immobilisation techniques, such as the use of sandbags and radiolucent positioning aids, to minimise unnecessary dose increases in paediatric WBCT examinations.

## Data Availability

The data associated with this will be made available by the corresponding author upon reasonable request.

## Abbreviations

CAP: Chest-abdomen-pelvic
CT: Computed tomography
CTDI_vol_: Volume computed tomography dose index
DLP: Dose length product
ED: Effective dose
GE: General Electric
ICRP: International Commission on Radiological Protection
IEC: International Electrotechnical Commission
MDCT: Multi-detector computed tomography
mSv: milli-Sievert
ROI: Region of interest
SNR: Signal-to-noise ratio
WBCT: Whole body computed tomography
